# Prevalence and Pattern of Morbidity, Febrile Illness, and Treatment-Seeking Behavior in a Tribal-Dominated District in Odisha, India: An Observational Study

**DOI:** 10.7759/cureus.58613

**Published:** 2024-04-19

**Authors:** Bhagirathi Dwibedi, Prajyoti Sahu, Nilam Somalkar, Anna S Kerketta, Hemant K Khuntia, Shantanu Kumar Kar

**Affiliations:** 1 Pediatrics, All India Institute of Medical Sciences, Bhubaneswar, Bhubaneswar, IND; 2 Clinical Research, Clinical and Epidemiology Division, Indian Council of Medical Research-Regional Medical Research Centre, Bhubaneswar, IND; 3 Public Health, Regional Office for Health and Family Welfare, Government of India, Bhubaneswar, IND; 4 Public Health, Clinical and Epidemiology Division, Indian Council of Medical Research-Regional Medical Research Centre, Bhubaneswar, IND; 5 Microbiology, Clinical and Epidemiology Division, Indian Council of Medical Research-Regional Medical Research Centre, Bhubaneswar, IND; 6 Medicine, Indian Council of Medical Research-Regional Medical Research Centre, Bhubaneswar, IND

**Keywords:** tribal, community volunteer, management, morbidity, febrile illness

## Abstract

Background

Tribal populations constitute a major portion of India’s total population, especially in the eastern and northeastern states. We lack comprehensive information on the community burden of general morbidity and febrile illness in tribal population-dominated areas, which is quite essential for the microplanning of healthcare expenditure and implementation. This study aimed to provide evidence on the prevalence and pattern of general morbidity and febrile illness at the community level as well as the treatment-seeking behaviour in a tribal-dominated area.

Methods

The study was undertaken as an observational study in the community setting; looking into seasonal cross-sectional evidence on period prevalence (two weeks) of morbidity and qualitative/semiquantitative information on treatment-seeking behaviour of the selected community during 2012 and 2013.

Result

This study involved 5541, 5482, and 5638 individuals during the rainy season 2012, winter 2012-13, and rainy season 2013 seasons, respectively, from 25 tribal villages of Odisha, India. A period prevalence (two weeks) of overall morbidities was shown to be 27.28% and 28.9% during the rainy seasons of 2012 and 2013, respectively, of which 13% and 11.5%, respectively, were febrile, with low prevalence (6.44% overall morbidity and 1.81% febrile illness) in the winter of 2012-13. It indicated inadequacy in skills of the village-level health staff, monitoring of supplies/logistics, and population awareness for early reporting of fever to healthcare providers at the community level.

Conclusion

The evidence provided by the study would be helpful in making public health plans in tribal settings and also highlighted the opportunity to improve tribal health status through community awareness, especially in areas and populations with limited health access.

## Introduction

Communicable diseases continue to be the major morbidity in countries like India; more so, in remote areas of tribal-dominated states like Odisha, Chhattisgarh, Jharkhand, and the North Eastern Region [1-3]. A major portion of childhood mortality is also contributed by acute respiratory infection (ARI) followed by diarrheal disorder [4,5] Though health indicators of the tribal populations are accepted to be lower than the national average of India, tribal-specific morbidity profile is not well documented. Reports from the North Eastern states of India indicate a high prevalence of febrile illness, and as per the National Family Health Survey, 1998-99 (NFHS-2), it was 38.5% (ranging from 28.4% in Assam to 41.2% in Meghalaya) compared to the national level of 29.5% [4,5]. High maternal and child mortality in these areas is attributed to undernutrition, infections, poor health awareness, and inaccessibility to health facilities due to inherent geographical difficulties, etc. [6]. Apart from these few reports, there is no systematic record of febrile illness or its aetiology available from tribal-dominated areas of the country. This raised the need to study the morbidity pattern in tribal populations, which would be useful in designing a realistic health promotion approach.

As most infectious illnesses present with fever, it logically becomes the most common presenting symptom and can be considered as the focus for morbidity assessment, especially for tribal populations that are limited by illiteracy. It is hypothesized that if febrile illness is focussed upon, a large chunk of morbidity can be taken care of and managed effectively at the village level. However, the current national programme doesn’t focus on a comprehensive management of febrile illnesses. Rather, it is diffused through multiple programmes like control programmes for malaria, ARI, and outbreak management. Lack of health-seeking behaviour, relative inaccessibility to health facilities in tribal-dominated areas, and perceiving fever as a casual symptom, curse of God, or misfortune are thought to limit early treatment, resulting in the severity of illness and death [5,7].

Anderson et al. reported poorer health and socioeconomic outcomes for Indigenous people across 23 countries after analysing data for 1997-2014. They documented low life expectancy at birth with a greater one-year difference in 15 populations compared to non-indigenous counterparts. This indicates high morbidity and poor health access for Indigenous tribal populations across the globe [8].

Mørch et al. reported that field studies on the aetiology of fever in India are scanty and limited by access to health facilities [9]. They observed malaria, dengue, and bacteremia in 17%, 16%, and 8% of patients (≥5 years), respectively, presenting with acute febrile illness from April 2011 to November 2012. However, the prevalence of acute febrile illness has not been studied. In a report by Kumar et al., a shortfall in healthcare infrastructure was observed in 18 states and three union territories of India with 27% of subcentres, 40% of primary health centres, and 31% of community health centres lacking adequate penetration of primary healthcare services [10]. The first comprehensive tribal health report proposal in India, made on the recommendation of the expert committee on tribal health (2013) in India, indicated less than 50% of the outpatient visits by tribal people were to public hospitals while more than two-thirds of hospital admissions were in government health facilities. This may imply low utilization of government health facilities for illnesses till these become severe enough for hospitalization [11].

It is thus observed that the available literature on the health scenario of tribal populations does not provide the overall burden of morbidity and febrile illness, although it gives an idea of certain diseases covered under national programs like malaria and tuberculosis as well as some situational analysis of health infrastructure. Hence, assessment of overall morbidity and health system functioning at the grassroots level would be essential to support the microplanning of public health.

We aimed to study the morbidity pattern in a tribal population, focusing on febrile illness and treatment-seeking behaviour of the community as well as health provider assessment. This will fill the deficit in evidence on morbidity prevalence in the tribal population and their treatment-seeking approaches, which can be useful in developing an alternate strategy framework to promote health services with community participation in remote tribal areas.

## Materials and methods

Study design

This study was conducted as a community-based cross-sectional survey to assess the morbidity prevalence during two prime seasons (rainy and winter) along with qualitative/semiquantitative assessment of the community health seeking and health delivery. The study was approved by the Institutional Ethics Committee, Indian Council of Medical Research-Regional Medical Research Centre, Bhubaneswar, Odisha, India (approval number: ECR/911/Inst/OR).

Study area and population

Rayagada, a tribal-dominated district in Odisha, India, was randomly selected for the study. It is situated between 190-200 North latitude and 230-800 East longitude. The average temperature of the area during summer remains about 29.39^o^C and in winter about 19.47^o^C, with an average annual rainfall of 1378.20 mm [12]. Of the district's total population, 55.8% belonged to scheduled tribes (2001 census), with Kandha and Saura as the major tribes [13,14]. The literacy rate of the district was 35.6% and the population thrives mostly on agriculture and forest product collection.

**Figure 1:**
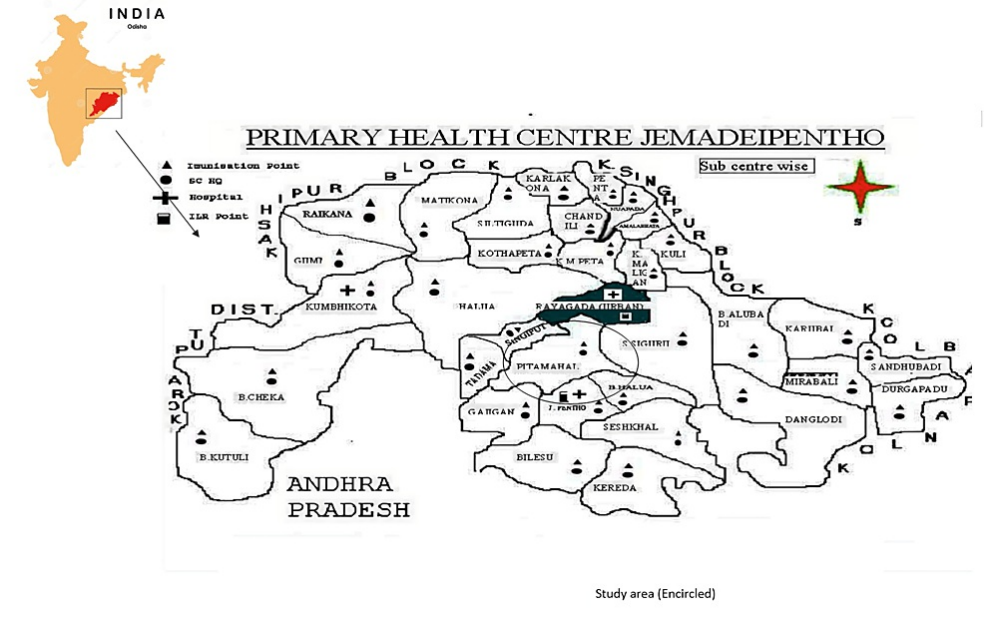
Map of study area under Jemadeipentho Primary Health Centre in Rayagada, Odisha, India Schematic Map (Not to scale) Image Credit: Authors

The study was conducted in three contiguous sub-centres namely Singiput, Tadama, and Pitamahal of one randomly chosen primary health centre (PHC), i.e., Jemadeipentha (Figure 1). Twenty-five villages under the PHC were included, to cover a population of around 5500 individuals without any age, gender, or household exclusion.

Sample size

Based on a fever prevalence of 7.4% as per the unpublished district annual survey report (2010-11) (Annual Morbidity Record: District Health Officials, Rayagada District, Odisha. March 2013) and the assumption of a higher prevalence in tribal-dominated villages, i.e., around 8.4%, the sample size was calculated to be 5573 for morbidity assessment, at 95% confidence level with 80% power of the study [15].

Morbidity and febrile illness survey

During two rainy seasons (July-August, 2012 and 2013) and one winter (December, 2012-January, 2013), we undertook a house-to-house survey after obtaining written informed consent from the head of the family and the adult participants/parents of children. Our team conducted a census enumeration of the population and filled up the questionnaires (See Appendix A) to record the demographic details and illness history of the individuals. The history of illness included febrile illness as well as afebrile disorders experienced within two weeks from the date of the survey. The individuals reporting some illnesses were requested to attend a central clinic set up in the village in a suitable location for evaluation by the team physician and necessary laboratory investigation for the common aetiologies. A syndromic approach for assigning diagnosis/aetiology of febrile/afebrile illness was used. Necessary laboratory investigations were performed to address common aetiologies. All the fever cases were tested for malaria, while a subset of cases with ARI (n=60) and diarrhoea (n=30) was tested for bacterial culture and viral reverse transcriptase-polymerase chain reaction (RT-PCR) as per feasibility.

Health-seeking behaviour and health system delivery were assessed by qualitative and semi-quantitative evaluations using questionnaires (See Appendix B) and a focus group discussion (FGD) approach covering the community and village-level health staff. staff. This included the perception of the population about the cause of fever, treatment preference (traditional medicine, government health system, etc.), knowledge and practices of village-level health staff (accredited social health activists (ASHAs), Anganwadi workers (AWW)), and community heads with regard to febrile illness and its management as well as the availability of logistics/supplies (rapid diagnostic kit (RDT) for malaria and fever medications). The questionnaire was pre-tested in the same population before use. Necessary cooperation was sought from the local health system. In each village, FGD was done with a group size varying from 16 to 25, where the village heads and adult male and female volunteers were included. The total community participants from 25 study villages were 610. FGD with village health staff covered 19 ASHAs, 23 AWWs, and three auxiliary nurses/midwives from the 25 study villages. Two ASHAs and one AWW could not participate in the FGD. 

The data were entered in Excel (Microsoft Corporation, Redmond, Washington, United States) and analyzed using IBM SPSS Statistics for Windows, Version 27.0 (Released 2020; IBM Corp., Armonk, New York, United States). Summarized data were expressed in the form of numbers, percentages/proportions, and means as appropriate. A p-value of <0.05 were considered significant for the statistical analysis comparing the proportion and means.

## Results

The survey for morbidity assessment covered 5541, 5482, and 5638 individuals including all ages and both genders during the rainy season of 2012, winter of 2012-13, and rainy season of 2013, respectively. During the rainy seasons, 1512 (27.29%) and 1629 (29.72%) individuals reported some morbidity in 2012 and 2013, respectively. However, during the winter season (2012-13), 353 (6.44%) individuals reported some morbidity. The details are given in Table 1.

**Table 1:** Morbidity and febrile illness recorded (two weeks period prevalence) in the surveyed population Data presented as frequency (percentage) P<0.015 between the prevalence of febrile illness during the rainy season of 2012 vs rainy season of 2013 and P < 0.0001 for rainy season of 2012 vs winter season of 2012-13; P<0.0001 between the prevalence of afebrile illness during the rainy season of 2012 vs rainy season 2013 and  P<0.0001 for rainy season of 2012 vs winter season of 2012-13 **ARI like allergic rhinitis, wheezing disorders, airway disease etc. presenting without fever; *ARI of upper/lower respiratory tract presenting with fever. ARI: acute respiratory infection

Sl No.	Pattern of Illness	Rainy season 2012 (July-August) (n=5541)	Winter season 2012-13 (December, 2012-February, 2013) (n=5482)	Rainy season 2013 (July-August) (n=5638)
1.	Febrile Illness	721 (13%)	99 (1.81%)	648 (11.5%)
a	Febrile ARI*	447 (8.06%)	70 (1.27%)	374 (6.63%)
b	Malaria	158 (2.85%)	8 (0.14%)	98 (1.73%)
c	UTI	71 (1.28%)	11 (0.2%)	39 (0.69%)
d	Others	45 (0.81%)	10 (0.18%)	137 (2.42%)
2.	Afebrile Illness	791 (14.27%)	254 (4.63%)	981 (17.39%)
a	Headache	189 (3.41%)	30 (0.55%)	294 (5.22%)
b	Diarrhoea/Dysentery	57 (1.03%)	19 (0.35%)	84 (1.49%)
c	ENT-related Morbidity	56 (1.01%)	6 (0.11%)	73 (1.3%)
d	Eye-related Morbidity	11 (0.2%)	12 (0.22%)	8 (0.14%)
e	Skin Infection	62 (1.12%)	33 (0.6%)	92 (1.63%)
f	Insect/Snake Bite	1 (0.02%)	1 (0.02%)	2 (0.03%)
g	Injury/Accident	11 (0.2 %)	8 (0.15%)	17 (0.3%)
h	ARI without fever**	135 (2.44%)	85 (1.55%)	118 (2.1%)
i	Body ache and Musculoskeletal pain	269 (4.85%)	60 (1.09%)	293 (5.2%)
j	Total	1512 (27.28%)	353 (6.44%)	1629 (28.9%)

The pattern of febrile illness was analyzed for the rainy season of 2012, which indicated febrile ARI (n=447) to be the most common (62%) followed by malaria (n=158; 22%) (Table 2). Among the samples tested (n=60) for bacterial/viral agents, febrile ARI was associated with bacterial infections in 45 (75%) cases *(Streptococcus pneumoniae* (n=20) 45%, *Haemophilus influenza *(n=7*)* 15.5%, *Staphylococcus* (n=7) 15.5%, viral infections in 12 (20%) samples (unclassified human coronaviruses (HCoV) 229E, NL63, HKU1, and OC43 (n=9) 15%, human parainfluenza viruses (n=3) 5%), and mixed infection in 10 (16.66%) cases. Bacterial pathogen for diarrhoea was isolated in nine (30%) (all *Escherichia coli*) of the stool samples (n=30) tested, while none revealed a viral agent. Among afebrile morbidities (N=791) in the rainy season of 2012, musculoskeletal pain (bodyache, myalgia, etc.) was most common (n=269; 4.85%), followed by headache (n=189; 3.41%).

**Table 2:** Pattern of febrile illness during rainy and winter seasons in the survey period (2012-13) Data presented as frequency (percentage) ARI: acute respiratory infection; UTI: urinary tract infection

Pattern of Febrile Illness	Rainy season 2012 (July-August) (N=721)	Winter season 2012-13 (December, 2012-February, 2013) (N=99)	Rainy season 2013 (July-August) (N=648)
Febrile ARI	447 (62%)	70 (70.70%)	374 (57.8%)
Malaria	158 (22%)	8 (8.08%)	98 (15.4%)
UTI	71 (9.84%)	11 (11.11%)	39 (6.01%)
Others	45 (6.24%)	10 (10.1%)	137 (21.14%)
Total febrile illness	721 (100%)	99 (100%)	648 (100%)

During rainy season 2013, of the total 648 febrile illness cases, ARI was commonest (n=374; 57.8%), followed by malaria (n=98; 15.4%). Similarly, during the winter season of 2012-13, ARI was commonest (n=70; 70.71%) among the 99 febrile illness cases, followed by malaria(n=8; 8%) (Table 2). Aetiological confirmation of bacterial and viral agents was not done for the rainy season of 2013 and winter 2012-13.

Age-wise prevalence of febrile illness reported during the rainy season of 2012 indicated higher prevalence in children below five years (17%) and adults above 45 years of age (45-60 years, 17.63%; >60 years, 28%) (Table 3).

**Table 3:** Prevalence of febrile illness in different age groups during the rainy season (July-August) of 2012 ^*^ % Calculated from the total population (N=5541); ^**^ % Calculated against census population (N) in the representative age group as denominator (n/Nx100) Data presented as frequency (percentage)

Age group	Census Population, N (%)*	Individuals with Febrile Illness, n (%) **
0-5 years	564 (10.18%)	96 (17.02%)
6-15 years	1308 (23.61%)	130 (9.94%)
16-30 years	1557 (28.1%)	109 (7%)
31-45 years	671 (12.11%)	86 (12.817%)
46-60 years	998 (18.01%)	176 (17.64%)
>60 years	443 (8%)	124 (28%)
Total	5541	721 (13.01%) (P=0.01)

Fever reporting and management undertaken by the village-level health staff comprising ASHAs, AWWs, and community volunteers were recorded and shown in Table 3. Our active survey indicated a 13% prevalence of febrile illness (period prevalence of two weeks) while the fever reported by the village health staff was 2.31%. In addition, we observed poor hand-washing practices as a surrogate of low preventive health practices (Table 4).

**Table 4:** Fever reporting and management at community level ^#^Fever prevalence was calculated by summating the reporting by community volunteers and ASHAs/AWWs; *Data based on qualitative/semi-quantitative assessment ASHA: accredited social health activist; RDT: rapid diagnostic test; AWW: Anganwadi worker

S. No.	Parameters	Observation
1	ASHAs acquired the skill of performing malaria RDT independently (N=19)	0 (0%)
2	ASHAs adequately trained on fever identification and management at village level (N=19)	0 (0%)
3	Reported fever prevalence (July-Aug 2012) by village health staff^#^ (N=5541)	128 (2.31%)
4	Fever reporting through our survey (July-Aug 2012) (N=5541)	593 (10.70%) (P<0.001)
5	Mean lag period in fever reporting from onset	4.5 days
6	Adequacy in supply of malaria RDT and drugs for fever management of fever cases (N=721)	238 (33%)^*^
7	School children adopting proper hand washing practice at mid-day meal	No record
8	Family members reporting acceptable handwashing practice with soap water before meals^*^(N=1230)	307 (25%)

Qualitative assessment (2012 and 2013) from participants (N=610) for treatment-seeking indicated that people preferred to receive health care from traditional healers (100%) and village health staff (ASHA and AWW) (n=488, 80%; 549, 90%) as the first level healthcare contact. Quacks or untrained practitioners (n=427, 70%), private doctors (n=244, 40%) and government health facilities (n=122, 20%) were the second-level preference.

Individual questionnaire survey (N=820; Rainy season, 2012: 721, and Winter 2012-13: 99) showed traditional healers (n=672, 82%), ASHA and AWW (n=508, 62%), quacks/untrained practitioners (n=623, 76%), PHC/CHC (n=344, 42%), and district hospital (n=213, 26%) as the routes of treatment used by the population. This indicated multiple sources or paths in seeking treatment.

Community perception (N=820; Rainy season, 2012: 721, and Winter 2012-13: 99) about the cause of fever revealed (a) blind beliefs such as bad spirit affecting children (n=508, 62%) and curse for violating taboos or social norms (n=393, 48%) and (b) hard physical labour (n=287, 35%).

Fever reporting to the health providers (N=820; Rainy season, 2012: 721, and Winter 2012-13: 99) within three days of onset was seen in 180 (22%) cases whereas it was between three to seven days in 229 (28%) and beyond seven days in 410 (50%) individuals. However, qualitative community assessment of different focus groups revealed an average delay of two to three days for seeking treatment for fever. The reported reasons for the delay in seeking fever treatment included ignoring fever considering it a low priority (n=590, 72%), home remedies were sufficient (n=672, 82%), waiting for the quack to visit the village (n=344, 42%), and apprehension towards treatment expenses (n=442, 54%). The quacks were reported to visit the villages on scheduled days on rotation and villagers waited for them as they were treated at their doorstep.

Interviews with ASHA (n=19) and AWWs (n=23) regarding fever management showed that 75% of ASHAs and 25% of AWWs have some knowledge of rapid diagnostic test (RDT) for malaria and use of paracetamol and antimalarials; 66.6% of ASHAs and 72.2% of AWWs were reported to have received one-time training on febrile illness management but no reorientation training was provided nor skill evaluation done to ensure RDT use. Of ASHAs and AWWs, 83.3% and 41.7%, respectively, reported having medicines in stock for fever management, and 75% of ASHAs and 58.3% of AWWs expressed that they attended all fever cases in the village. However, 75% of ASHAs and 81.8% of AWWs suggested that retraining on fever management and regular and adequate supply of logistics would improve their service.

## Discussion

Our study has shown morbidity prevalence (period prevalence over two weeks) of 27.28% and 28.9% during rainy seasons of the years 2012 and 2013, respectively, in a tribal setting which included 13% and 11.5% febrile illness, while it was low (1.81%) during winter. Infectious illnesses and musculoskeletal symptoms were higher in rains, which might be due to seasonal pathogen exposure and increased physical labour for agriculture, respectively. Such seasonal difference was also reported for febrile illness from a study by Youssef et al. involving 1375 residents of three villages in Somalia which showed a period (14 days) prevalence of self-reported fever between 4.8% (March) and 0.6% (August) [15]. In their study, the majority of fever cases (84.4%) were associated with cough, running nose and sore throat and with a reporting time of 5.4 days, only 37.5% were managed in a formal health care facility and none tested for malaria.

In the current study, ARI was noted to be the most common (62%) cause of febrile illness, but second was malaria (22%). Crump et al. also reported acute febrile illness as the common cause of morbidity and mortality in children and adults in middle- and low-income countries [16]. Fares et al. stated that seasonal variation in incidence of infectious diseases is likely and might be influenced by population behaviours and environmental factors affecting pathogen abundance [17].

The age-wise prevalence of febrile illness during July-August 2012 indicated a higher prevalence in children under five years (17.02%) and adults above 45 years (45-60 years: 17.64% and >60 years: 28%). This indicates greater prevalence in the extremes of ages as well as the active population above 45 years.

The study by Raushan and Acharya revealed about 14% prevalence of acute illness of any kind over 30 days among the ST population compared to 19% in non-ST communities [2]. Around 90% of acute illnesses were fever followed by ARI (28%) and diarrhoea (17%) in the tribes.

As per a WHO compilation (2013), fever incidence in rural tropical areas was estimated at up to 10 per person-years in <5 years of age and four per person-years in older individuals [18]. However, these estimates were widely varied according to location, fever definition, season, and survey methods used in different studies [19]. Truly, there are few reliable data on the actual burden of febrile illness in tropical or subtropical tribal areas.

An ethnographic study (2000) from the Philippines regarding treatment paths for fever (malaria) showed 26 treatment paths. It identified self-treatment with western medicine and clinic consultation at almost similar frequencies, though for children, clinic consultation was more [20]. Our present study from a tribal district of Odisha indicated traditional healers to be the first preference, though 80-90% co-opted advice from village-level government health staff. Untrained treatment providers (usually identified as quacks) and private practitioners were preferred over government-run PHCs and district hospitals as the second-level contact or referral.

Prabhakar and Manoharan opined that tribal populations generally have poor health outcomes, often because of a healthcare delivery system that does not cater to their actual needs [7]. However, they noted a positive behaviour of increased confidence in allopathic medicine after the implementation of the health system and emphasized long-term educational initiatives for critical change.

The Third National Family Health Survey report (NFHS-3) from India (2005-06) indicated an average fever prevalence of 15% among children under five years during two weeks but 29% with fever were not taken to a health facility/provider, which was more often for rural than urban and for girls than boys [5]. Treatment seeking was further low for scheduled tribe children [5]. This may point towards poor utilization of health services, especially in rural and tribal settings. Out of the total surveyed population of 5541 during the rainy season of 2012, in our study, the number of fever cases recorded was 721 (13%). However, the number of cases reported to village health staff was only 128 (2.31%). Hence, 10.7% were missed from the official reporting in the routine health survey and is reflected as additional fever reporting through our survey team (Table 4).

Prabhakar and Manoharan opined on a need for the implementation of models with social-cultural disparities among tribes factored in [7]. They demonstrated a tribal health initiative model on the Malayali and Lambadi populations in a southern Indian district. The model had a base hospital as a nodal point armed with local health workers, who were tribal women with education up to eighth standard and 1.5-year hospital-based training. Illiterate tribal women of childbearing age acted as health auxiliaries. This model explored an opportunity to develop alternative models for tribal settings in the country. Balgir also highlighted the need to capacitate the traditional healers by linking them with modern health institutions as well as the need for health centres to be fully equipped with round-the-clock availability of medicines [3]. Hence, different studies indicated a need for special attention to the management of febrile illness and common morbidities in underdeveloped populations like scheduled tribes.

Summarizing our observations, a high prevalence of febrile as well as afebrile illness is emphasized, the major portion of which is missed from reporting and unattended by the government health system at the first point of contact. Population reliance on the health system/facilities seems to be inadequate, so also the preparedness of public health departments in optimizing the supply of logistics for diagnosis and treatment of illnesses encountered at the village level. Strengthening the surveillance of acute febrile illness at least in a syndromic mode is recommended, which will provide a real-time estimation of the healthcare need. This will help to bridge the gaps in the supply chain, health manpower deployment, and morbidity management of the outreach tribal population.

Against this background, we suggest that an improved implementation strategy be looked into for strengthening the current health system with a multipronged approach covering the training of health workers at the village level, improving awareness through community participation, linking community volunteers, and monitoring logistics flow or supply of diagnostics and drugs. Evidence also supports our proposition for an integrated community case management strategy involving community volunteers, as studied in rural Uganda [21]. Community volunteers were also shown to be effective in malaria case management in rural Odisha [22]. Though the job assigned to the volunteers was addressing only chloroquine distribution to presumptive malaria cases, the feasibility of the model and acceptance to village-level volunteers was evident. This can form a strong base while designing a public health model to address all febrile illnesses comprehensively in a syndromic mode.

Limitations of the study

Our study was limited by a deficiency in complete laboratory investigation for all the subjects that would have provided additional information on the aetiology of febrile and afebrile illnesses recorded during the period. Hence, we suggest a detailed study on aetiological evaluation which will help in providing inputs towards specific requirements of diagnostic kits and medications.

## Conclusions

This study showed a high period prevalence (two weeks) of overall morbidities at 27.28% and 28.9% during the rainy seasons of 2012 and 2013, respectively, of which 13% and 11.5%, respectively, had a febrile illness, with low prevalence in winter. It also indicated inadequacy in skills of the village-level health staff, monitoring of supplies/logistics, and population awareness for early reporting of fever to treatment providers at the community level, thereby prolonging the illness. This evidence would be helpful to make public health plans in tribal settings and also provide an opportunity to think of improving tribal health status through community awareness, especially in areas and populations with limited health access and addressing the gaps in health delivery skills of village-level health staff.

## Figures and Tables

**Figure 2:**
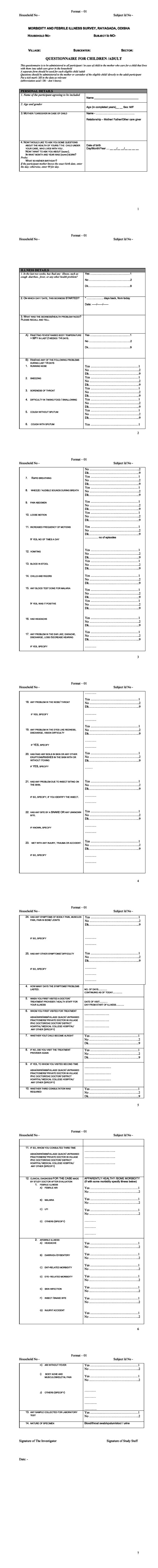
Morbidity and febrile illness survey, Rayagada, Odisha - Questionnaire format 1

**Figure 3:**
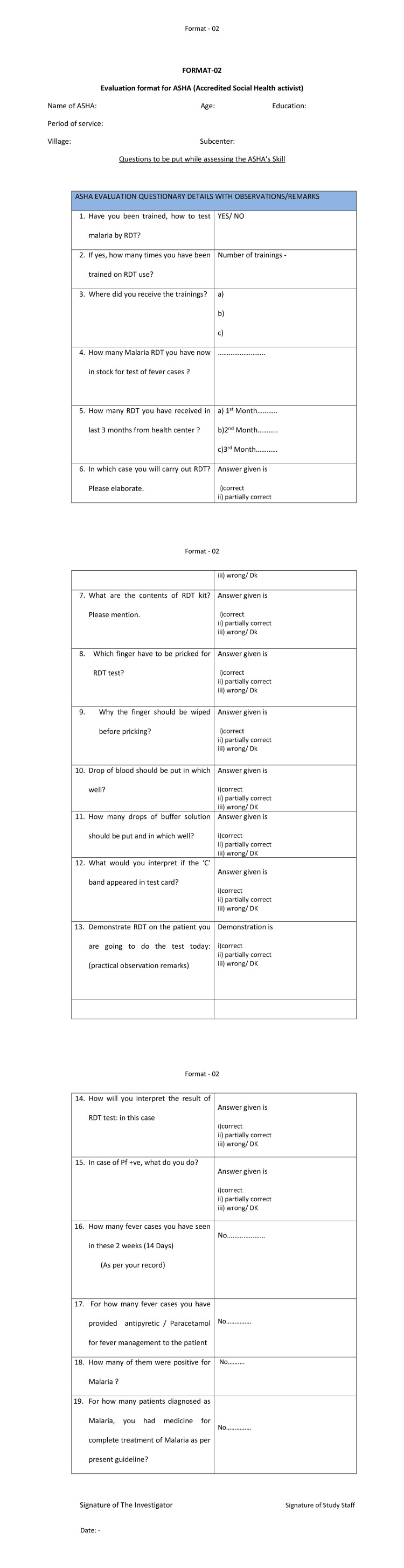
Evaluation format for community health workers - Questionnaire format 2

## Appendices

Appendix A

Appendix B
